# Arthroscopic one-stage management of diffuse shoulder pigmented villonodular synovitis with concomitant full-thickness rotator cuff tear and acromial bone erosion: a rare case report

**DOI:** 10.3389/fsurg.2026.1792575

**Published:** 2026-05-18

**Authors:** Chenglong Wang, Xiaoping Zhou, Jialin Zhuang, Yang Ding, Fulin Tang

**Affiliations:** Upper Extremity Department, Mianyang Orthopaedic Hospital, Mianyang, Sichuan, China

**Keywords:** bone defect, case report, pigmented villonodular synovitis, PVNS, rotator cuff tear, shoulder arthroscopy

## Abstract

The coexistence of diffuse shoulder pigmented villonodular synovitis (PVNS), full-thickness rotator cuff tear, and PVNS-induced focal acromial bone erosion is extremely rare, with no prior reports documenting this complete pathological triad, which poses unique diagnostic and therapeutic challenges. We report a 52-year-old male with a three-month history of progressive right shoulder pain, swelling, and functional limitation. Radiographs showed cortical irregularity of the acromion, while MRI revealed extensive synovial proliferation with low signal on T1/T2 sequences, suggestive of PVNS, and a full-thickness supraspinatus tendon tear. The patient underwent single-stage arthroscopic synovectomy, rotator cuff repair using a double-row suture bridge technique, and debridement of a small acromial bone defect. Histopathology confirmed diffuse-type PVNS. Preoperative ASES and VAS scores were 38.2 and 6, respectively, improving to 75.8 and 2 at three months, and to 92.5 and 1 at five months post-surgery, with satisfactory early functional recovery. This case highlights that PVNS should be included in the differential diagnosis of persistent shoulder monoarthritis, even in patients with typical rotator cuff tear signs, especially when accompanied by atypical bone lesions on imaging. One-stage combined arthroscopic complete synovectomy, anatomical rotator cuff repair, and bone erosion debridement is a feasible and effective minimally invasive strategy for managing this complex pathological combination. The early clinical outcomes are encouraging, however, given the short follow-up, these early outcomes should be interpreted cautiously, and long-term surveillance is required.

## Introduction

Pigmented Villonodular Synovitis (PVNS) is a benign, locally aggressive tumor-like proliferation of the synovium, characterized by villous and nodular overgrowth with hemosiderin deposition (1). It typically occurs in adults aged 20–50 years, with a slight female predominance. The knee joint is most frequently involved (accounting for approximately 80% of cases), followed by the hip and ankle (2). Shoulder involvement constitutes less than 5% of all PVNS cases, rendering it a rare pathology within the shoulder (3). Its clinical presentation is often insidious, with symptoms including pain, swelling, stiffness, and recurrent hemorrhagic joint effusions. If left untreated, the proliferative synovium can erode cartilage and bone, leading to significant osteoarticular destruction and secondary osteoarthritis (4). The coexistence of PVNS with other common shoulder pathologies, such as a full-thickness rotator cuff tear, is rarely documented in the literature (5, 6). Furthermore, the presence of concomitant bone defects adds considerable complexity to both diagnosis and surgical management. The primary treatment for diffuse-type PVNS is complete synovectomy, aimed at reducing the substantial risk of local recurrence, which ranges from 14% to 50% (7). While open synovectomy has been the traditional approach, arthroscopic techniques are gaining popularity due to advantages such as improved visualization, smaller incisions, less postoperative pain, and faster rehabilitation (8). However, in the shoulder, achieving complete arthroscopic synovectomy is technically demanding, especially in the context of concurrent rotator cuff tears. While isolated shoulder PVNS or PVNS combined with rotator cuff tear has been reported in previous literature, the concurrent presence of PVNS-induced focal acromial bone erosion, even if small in size, represents a more advanced locally aggressive stage of the disease, and this three-pathology combination has not been previously described. Therefore, we present in detail the diagnostic workup and successful one-stage arthroscopic management of a patient with this rare pathological triad, aiming to supplement the limited clinical evidence and provide standardized treatment references for this specific clinical scenario.

## Case report

**Figure 1:**
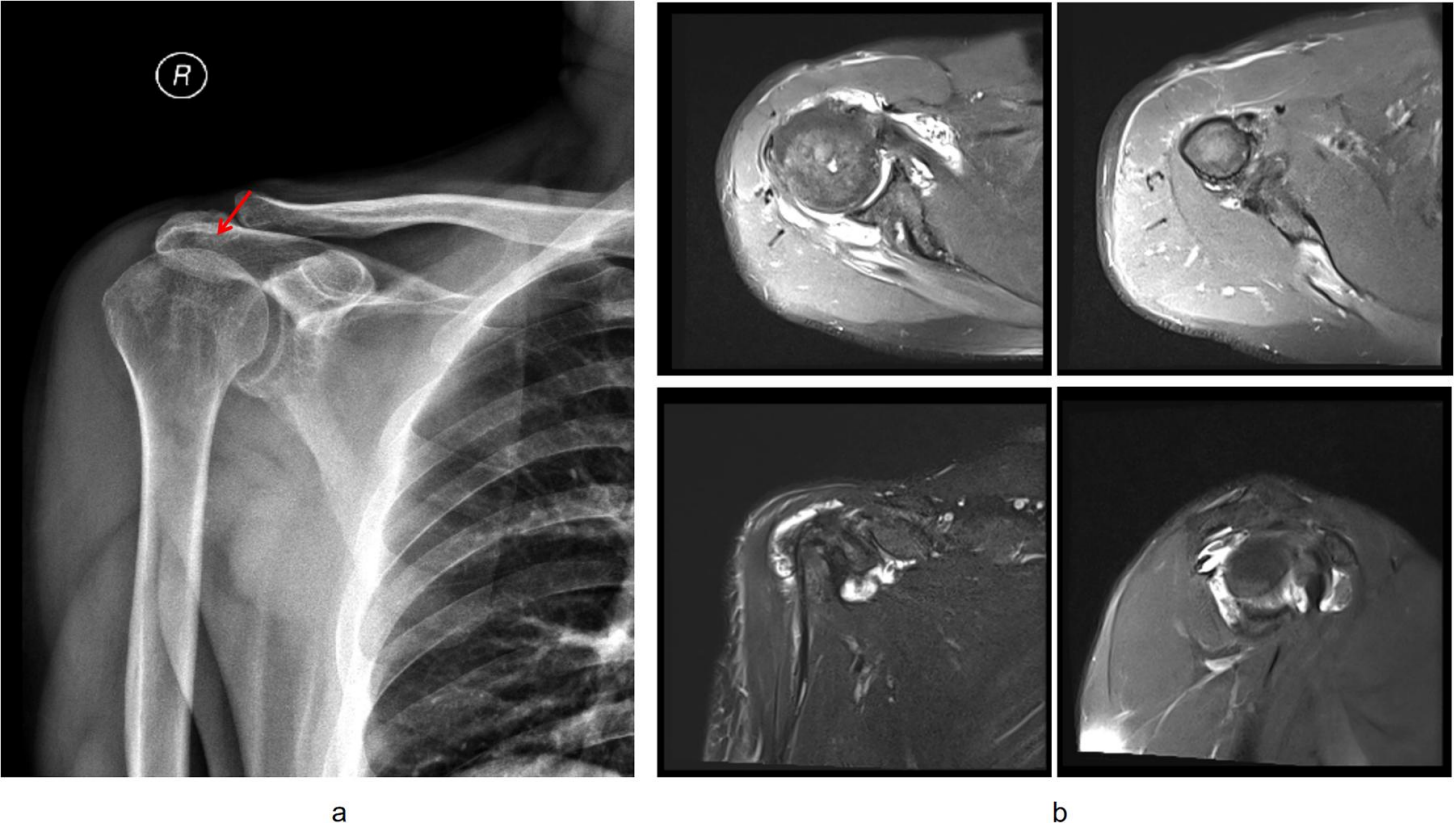
**(a)** anteroposterior radiograph of the right shoulder demonstrating cortical irregularity and subtle bone density changes involving the undersurface of the acromion (red arrow), suggestive of focal bone erosion; **(b)** coronal T2-weighted fat-suppressed MRI showing extensive hypointense synovial proliferation within the glenohumeral joint and subacromial space, consistent with PVNS, along with a full-thickness supraspinatus tendon tear.

A 52-year-old male presented to our hospital with a three-month history of pain and limited mobility in his right shoulder, which began following a period of strenuous right upper limb activity. Previous treatment at another clinic, involving physical therapy and medication, had provided no symptomatic relief. Positive physical examination findings upon presentation included a positive Empty Can (Jobe) test, Drop Arm test, painful arc sign, and External Rotation Lag sign. His pre-operative Constant-Murley Score was 30 (Pain: 5, Activities of Daily Living: 8, Range of Motion: 12, Strength: 5). The pre-operative ASES score was 38.2 (Pain: 20/50, Function: 18.2/50), and the VAS pain score was 6. Radiographs revealed cortical irregularity of the right acromion (Figure 1a). Magnetic Resonance Imaging (MRI) demonstrated extensive synovial proliferation exhibiting characteristic low signal intensity on both T1- and T2-weighted sequences, suggestive of PVNS, alongside a full-thickness tear of the supraspinatus tendon (Figure 1b). Pre-operative hematological tests, including erythrocyte sedimentation rate, rheumatoid factor, and C-reactive protein, were within normal limits.

**Figure 2:**
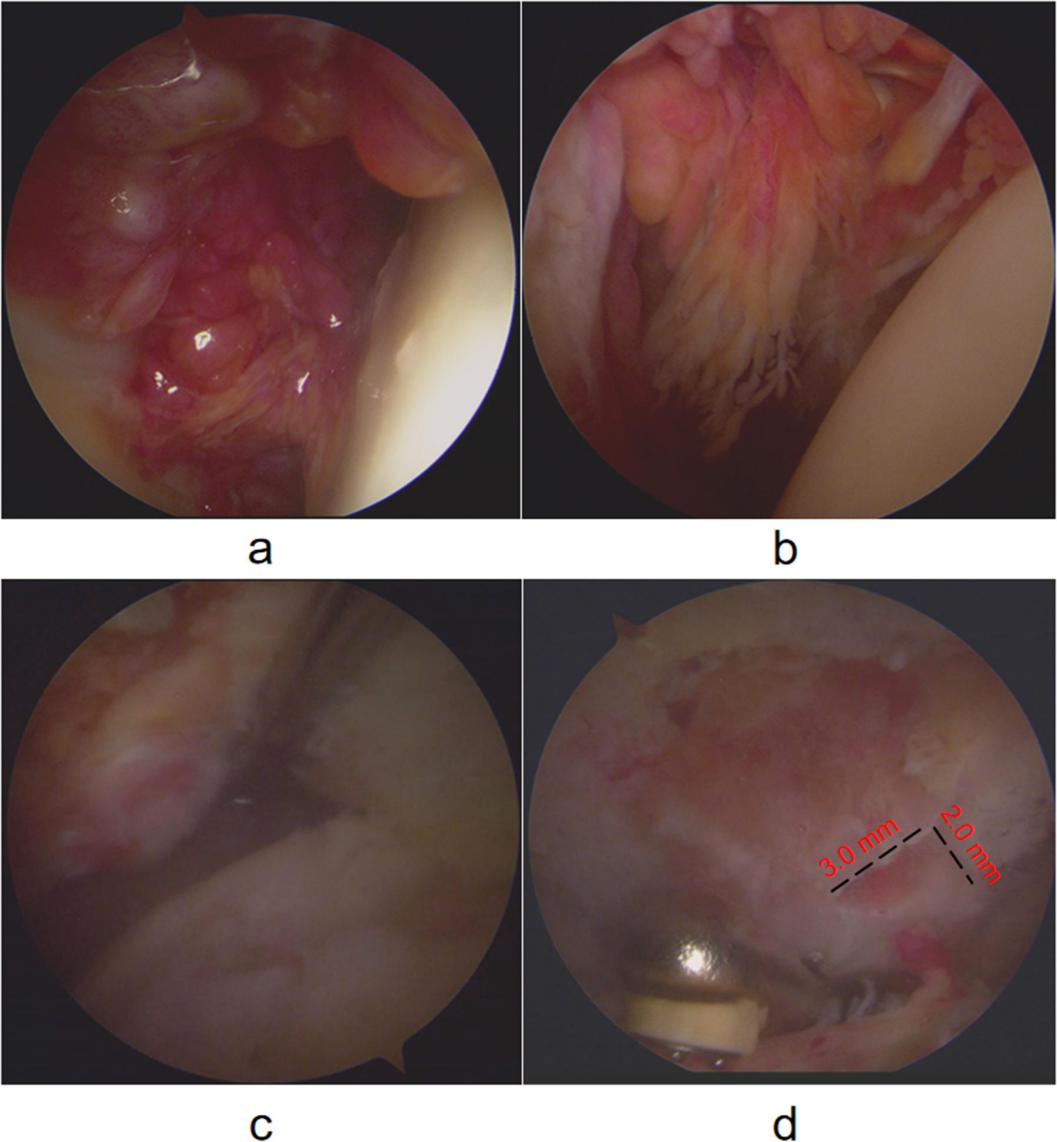
**(a,b)** arthroscopic views from the posterior portal showing diffuse, dark-brown pigmentation and villous hypertrophy on the synovial surface of the glenohumeral joint, characteristic of PVNS; **(c)** view from the lateral portal revealing a full-thickness tear of the supraspinatus tendon at its insertion on the greater tuberosity; **(d)** subacromial view showing a focal bone erosion lesion (approximately 3 × 2 mm) on the undersurface of the acromion with irregular, non-sclerotic margins.

**Figure 3:**
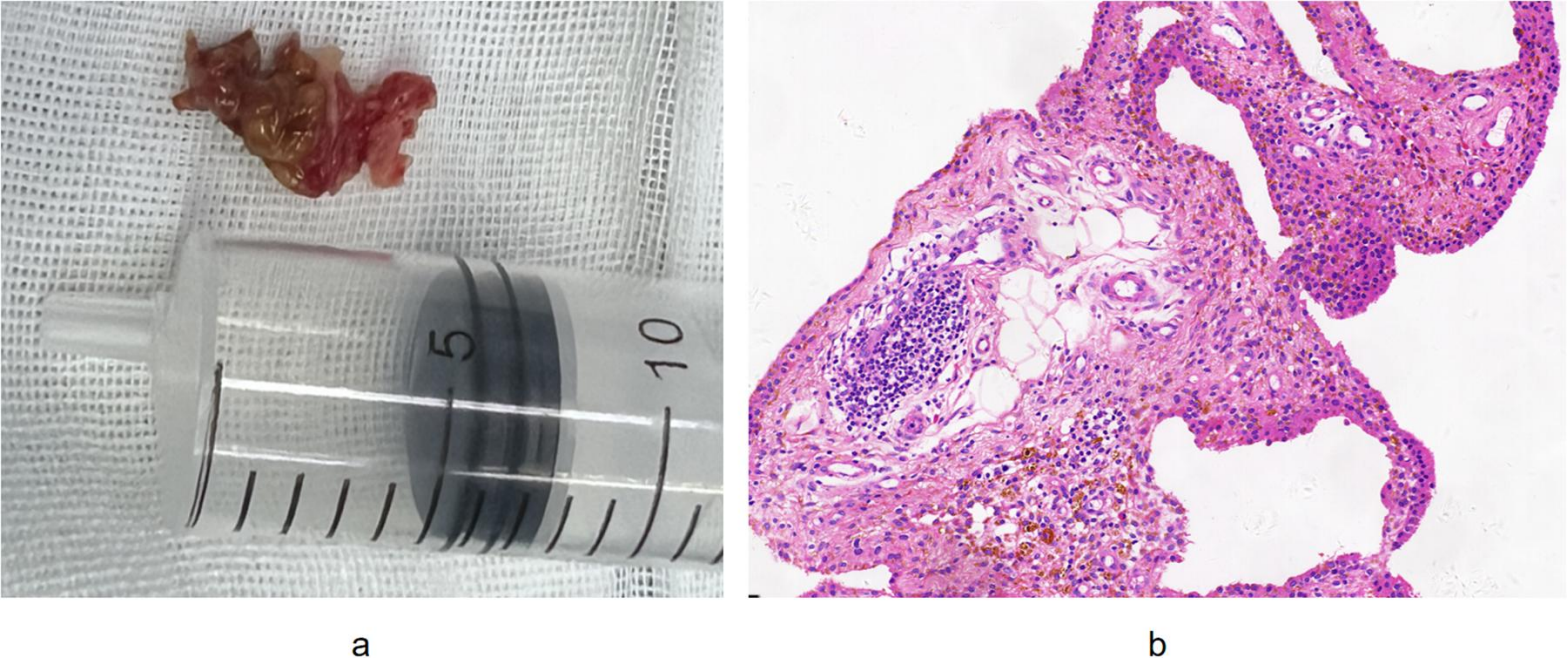
**(a)** gross photograph of the resected synovial tissue showing the characteristic brownish-yellow pigmentation and villonodular architecture; **(b)** hematoxylin-eosin stained section (original magnification × 100) revealing chronic synovitis with marked synovial hyperplasia, hemosiderin deposition, and focal fibrinous necrosis.

**Figure 4:**
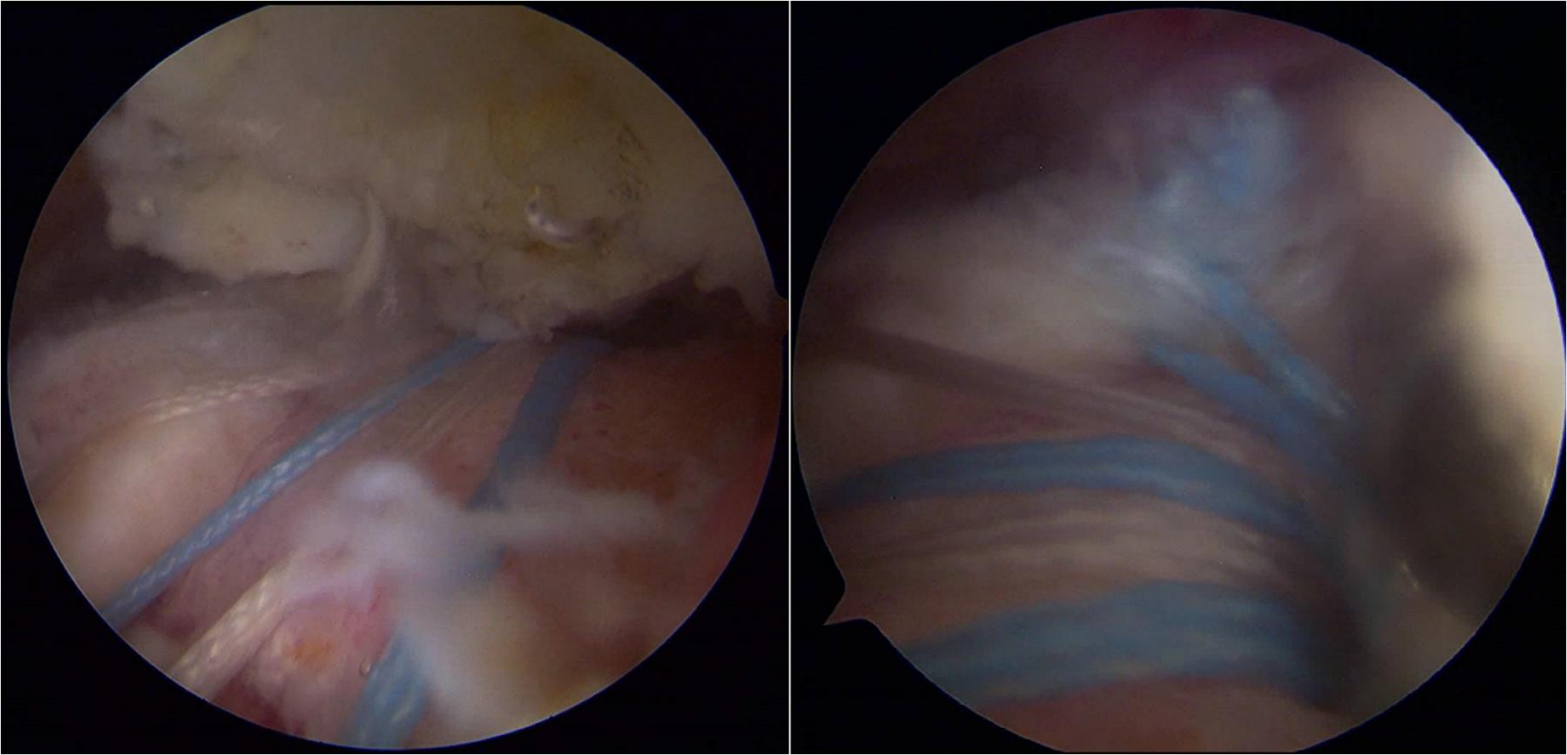
Arthroscopic view from the lateral portal demonstrating the final repair of the supraspinatus tendon using a double-row suture bridge technique, with excellent footprint coverage and stable fixation.

The treatment plan involved arthroscopic surgery to repair the torn rotator cuff and perform a synovectomy. The patient was placed in the beach-chair position. A standard posterior portal was first established for diagnostic arthroscopy. Thereafter, additional anterior and lateral portals were created as needed to facilitate access to the glenohumeral joint and subacromial space. Intraoperative arthroscopic visualization revealed extensive brownish pigmentation on the synovial and other intra-articular surfaces (Figures 2a,b). A thorough synovectomy was performed using a combination of radiofrequency ablation and motorized shaver. Representative samples of the hyperplastic synovium were harvested with a small grasping forceps and sent for histopathological examination. The goal was to resect all visible pathological tissue while preserving healthy structures (Figure 3a). Intraoperative inspection of the acromion identified a focal bone erosion lesion (approximately 3 × 2 mm) with exposed cancellous bone and irregular, non-sclerotic margins on the undersurface of the acromion (Figure 2d). The lesion was directly adjacent to the hyperplastic pigmented synovium in the subacromial space, consistent with PVNS-induced bone invasion rather than mechanical impingement-related degenerative change. Given its limited size and depth, which did not compromise glenohumeral joint stability or the integrity of the rotator cuff repair, the decision was made to thoroughly debride the lesion with a burr to remove all potentially invaded synovial tissue and create a smooth bone surface, eliminating residual nidi for PVNS recurrence. No bone grafting or additional stabilization was performed, as the lesion was deemed biomechanically insignificant. Subsequent arthroscopic visualization revealed a full-thickness tear of the supraspinatus tendon (Figure 2c). The full-thickness supraspinatus tendon tear was repaired using a double-row suture-bridge technique. Specifically, three double-loaded suture anchors were inserted medially at the articular margin of the footprint, and one lateral row anchor was used to secure the suture tails over the tendon, achieving compression and stable fixation. The repair was confirmed to be robust and provided good tendon coverage of the footprint (Figure 4). The patient's shoulder pain improved immediately post-operatively, and the right shoulder was immobilized in abduction. Histopathological analysis confirmed chronic synovitis with synovial hyperplasia and focal fibrinous necrosis, consistent with PVNS given the clinical context (Figure 3b).

**Figure 5:**
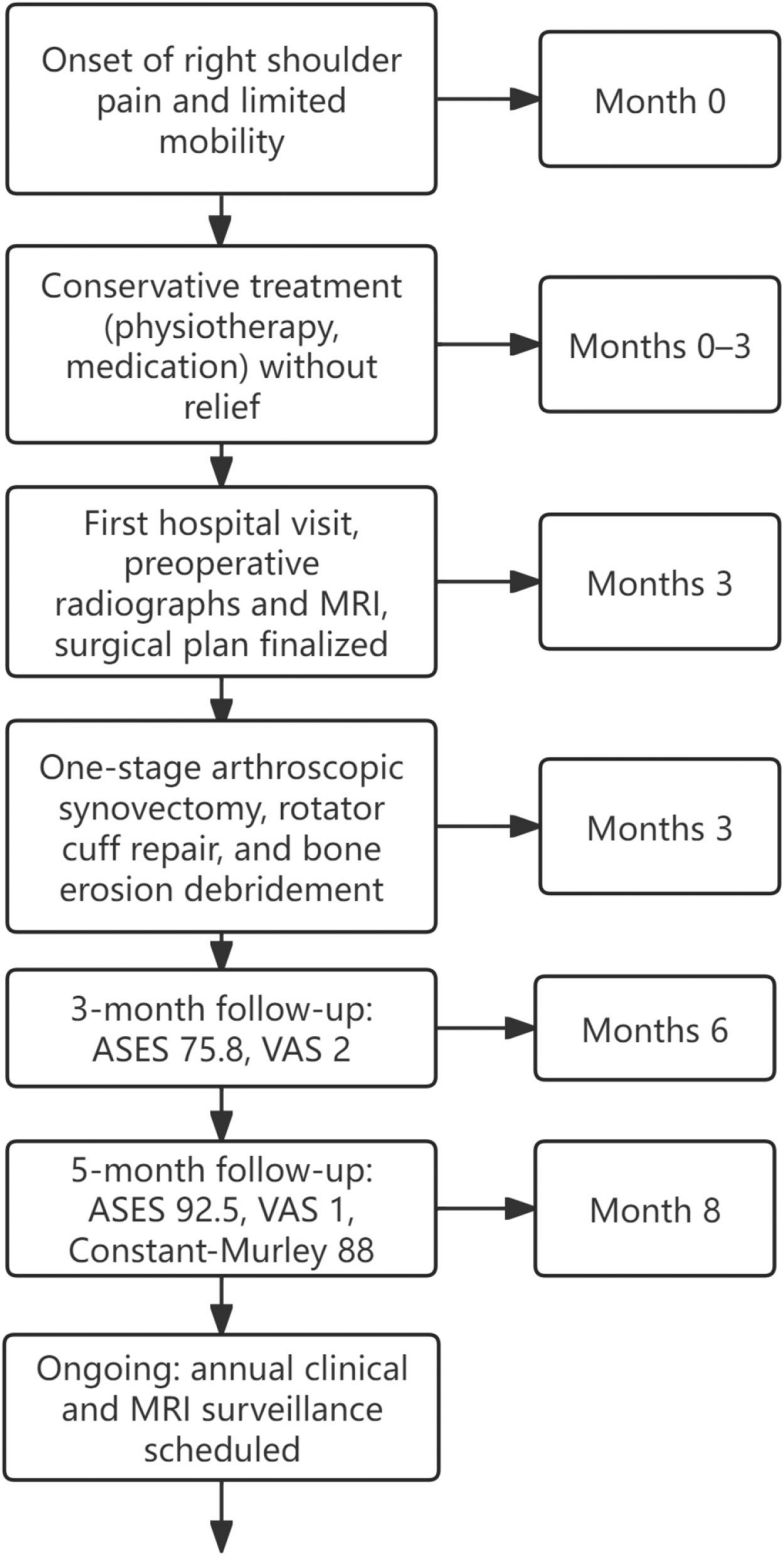
Clinical timeline summarizing the key events from symptom onset to the latest follow-up. Month 0: onset of right shoulder pain and limited mobility; Months 0–3: conservative treatment (physiotherapy, medication) without relief; Month 3: first hospital visit, preoperative radiographs and MRI, surgical plan finalized and one-stage arthroscopic synovectomy, rotator cuff repair, and bone erosion debridement; Month 7 (3-month follow-up): ASES 75.8, VAS 2; Month 9 (5-month follow-up): ASES 92.5, VAS 1, Constant-Murley 88; Ongoing: annual clinical and MRI surveillance scheduled.

Post-operatively, the patient initiated a gradual functional rehabilitation program [including phase I (weeks 1–4): immobilization in abduction; phase II (weeks 5–8): passive and active-assisted range of motion exercises; phase III (week 9 onwards): progressive strengthening]. At the three-month post-surgical follow-up, the patient demonstrated significant improvement in pain and shoulder function, with a Constant-Murley Score of 70 (Pain: 13, Activities of Daily Living: 14, Range of Motion: 23, Strength: 20). The ASES score improved to 75.8 (Pain: 40/50, Function: 35.8/50), and the VAS pain score decreased to 2. At the five-month follow-up, the patient's shoulder function had recovered to near-normal levels, with an ASES score of 92.5 (Pain: 48/50, Function: 44.5/50), a VAS pain score of 1, and a Constant-Murley Score of 88 (Pain: 15, Activities of Daily Living: 18, Range of Motion: 30, Strength: 25). The patient had resumed daily activities and light physical exertion without discomfort, reporting high satisfaction with the surgical outcome. A detailed clinical timeline is presented in Figure 5.

Given the potential risk of PVNS recurrence, adjuvant radiotherapy was considered as a therapeutic option following literature review. However, as our institution lacks radiation oncology facilities, the patient was advised to seek consultation at a specialized center. He declined further intervention, expressing satisfaction with the surgical outcome and preferring conservative monitoring. His decision was fully respected.

## Discussion

This report describes an extremely rare case of diffuse-type pigmented villonodular synovitis (PVNS) of the shoulder, presenting with a complete pathological triad of diffuse synovial proliferation, full-thickness rotator cuff tear, and focal acromial bone erosion. To our knowledge, this specific combination of pathologies has not been previously documented in the literature. The patient achieved satisfactory early pain relief and functional recovery via one-stage arthroscopic comprehensive management, including complete synovectomy, anatomical rotator cuff repair with a double-row suture bridge technique, and thorough debridement of the PVNS-related bone erosion. This case expands the current understanding of the natural history of shoulder PVNS, and provides a feasible, minimally invasive treatment strategy for this rare complex clinical scenario.

### Pathological significance of the triad: diffuse PVNS, rotator cuff tear, and acromial bone erosion

The core novelty of this case lies in the identification of a complete, pathologically linked triad of shoulder PVNS, full-thickness rotator cuff tear, and acromial bone erosion, rather than three isolated incidental findings. We hypothesize a possible pathological progression sequence based on intraoperative and imaging observations in this case, though confirmation requires further evidence.

First, the primary pathological lesion is diffuse PVNS of the glenohumeral joint. The hyperplastic, hemosiderin-laden synovial tissue exhibits inherent locally invasive properties, which directly invades the supraspinatus tendon at its insertion site, destroys the collagen fiber structure, and eventually leads to a full-thickness rotator cuff tear. This pathological process explains why chronic synovitis caused by PVNS can result in rotator cuff tear, which cannot be simply attributed to degenerative changes in middle-aged patients. Second, the full-thickness rotator cuff tear forms a pathological channel between the glenohumeral joint and the subacromial space, allowing the proliferative synovial tissue to extend into the subacromial bursa. The extended pathological synovium directly contacts and invades the undersurface of the acromion, leading to focal bone erosion, even in the absence of severe mechanical impingement.

Notably, although the acromial bone erosion is only 3 × 2 mm in size and does not require bone grafting or structural reconstruction, it is by no means a clinically insignificant incidental finding. On the contrary, it has critical pathological and clinical implications: 1) It is direct imaging and intraoperative evidence of the locally aggressive nature of diffuse PVNS, marking a more advanced disease stage compared with previously reported shoulder PVNS cases with only rotator cuff tear and no bone involvement; 2) The bone erosion lesion may harbor residual pathological synovial tissue, which is a potential nidus for postoperative recurrence, necessitating thorough debridement during surgery even for small lesions; 3) The presence of PVNS-related bone erosion indicates a higher risk of local recurrence, which requires more rigorous long-term clinical and imaging follow-up, and earlier consideration of adjuvant radiotherapy if necessary. While the observed spatial and temporal relationship among the three lesions suggests a possible pathological continuum—with PVNS as the primary driver—this interpretation remains speculative based on a single case and should be validated in future studies.

### Literature review

Shoulder PVNS is a rare clinical entity, accounting for less than 5% of all PVNS cases (3). The coexistence of shoulder PVNS and rotator cuff tear is even rarer, with only two small case series reported in the literature to date. To clearly highlight the innovations and clinical relevance of this case compared with previous reports, we systematically compared the core clinical characteristics of all relevant published cases with the present case in Table 1.

**Table 1:** Comparison of clinical characteristics between the present case and previously reported shoulder PVNS cases with rotator cuff tear (recurrence data were extracted from the original reports where available; however, most studies did not systematically report recurrence rates, and follow-up durations varied considerably, limiting direct comparison).

Study	Year	Number of Cases	PVNS Type	Concomitant Rotator Cuff Tear	PVNS-Related Bone Erosion	Surgical Strategy	Standardized Functional Outcome	Follow-up Duration	Recurrence Rate
Gumina et al. (5)	2013	3	Diffuse	Massive, irreparable	None reported	Arthroscopic synovectomy + debridement only	No standardized ASES/Constant-Murley score reported	12–36 months	Not stated
Chiang et al. (6)	2009	2	Diffuse	Massive	None reported	1 case: synovectomy + debridement; 1 case: partial rotator cuff repair	No standardized ASES/Constant-Murley score reported	24–36 months	0% at reported follow-up
Li et al. (8)	2022	12	Diffuse/Localized	None	None reported	Arthroscopic synovectomy alone	Mean ASES score improved from 42.3 to 81.5 at final follow-up	Mean 38.2 months	Not stated
Present case	2025	1	Diffuse	Full-thickness, repairable	Focal acromial bone erosion	One-stage arthroscopic complete synovectomy + double-row suture bridge anatomical rotator cuff repair + bone erosion debridement	ASES score improved from 38.2 to 92.5 at 5 months; Constant-Murley score improved from 30 to 88 at 5 months	5 months (ongoing annual follow-up scheduled)	Not assessable (5-month follow-up only)

Based on this systematic comparison, we have clearly defined three core innovations and clinical guiding values of this case that have not been reported in previous literature: To our knowledge, this is the first reported case of shoulder PVNS presenting with the complete triad of synovial proliferation, full-thickness rotator cuff tear, and direct bone erosion of the acromion. While two prior case series have documented PVNS with concurrent rotator cuff tear (5, 6), neither reported PVNS-related bone involvement. The primary novelty of the present case therefore lies in: the documentation of focal acromial bone erosion as a marker of locally advanced PVNS, and the first description of one-stage arthroscopic management addressing all three pathologies simultaneously.

All previously reported cases of shoulder PVNS combined with rotator cuff tear only underwent palliative synovectomy and debridement, or partial rotator cuff repair at most. In contrast, this case is the first to achieve one-stage comprehensive management of all three pathological lesions via arthroscopic surgery, including complete synovectomy of both the glenohumeral joint and subacromial space, anatomical rotator cuff repair with a double-row suture bridge technique, and thorough debridement of the bone erosion lesion. The early functional outcomes of this case are superior to those of previous reports using synovectomy alone, confirming that one-stage anatomical rotator cuff repair can be safely performed simultaneously with complete synovectomy for repairable tears, and can significantly improve early functional recovery without increasing the risk of recurrence.

We have summarized three key clinical principles from this case for the diagnosis and treatment of similar rare scenarios: 1) Preoperative MRI is mandatory for patients with chronic shoulder monoarthritis to rule out PVNS and other rare synovial lesions, even in the presence of typical rotator cuff tear signs; 2) For PVNS combined with repairable rotator cuff tear, one-stage anatomical repair should be actively pursued, rather than only palliative debridement, to restore the biomechanical balance of the shoulder joint and improve long-term functional prognosis; 3) Even for small PVNS-related bone erosion lesions, thorough debridement is required during surgery to eliminate residual recurrence nidus, and more rigorous long-term follow-up is necessary.

### Key clinical considerations for diagnosis and surgical management

MRI plays a pivotal role in the preoperative diagnosis of shoulder PVNS, which is consistent with previous reports (9). The characteristic low signal intensity on both T1- and T2-weighted sequences, caused by hemosiderin deposition in the proliferative synovium, is the key imaging marker for the diagnosis of PVNS. In this case, MRI clearly delineated the extent of synovial proliferation and the full-thickness rotator cuff tear, which provided critical guidance for the surgical plan. However, the small focal acromial bone erosion was not detected on preoperative MRI, which reminds clinicians that intraoperative comprehensive exploration of the subacromial space is mandatory, especially in patients with full-thickness rotator cuff tears, to avoid missing PVNS-related bone lesions.

In terms of surgical approach, arthroscopic surgery has significant advantages over open surgery for the management of shoulder PVNS, which is consistent with the conclusions of previous studies (8, 10). Arthroscopy provides superior magnification and visualization of the posterior and inferior recesses of the glenohumeral joint, which facilitates complete resection of all pathological synovial tissue—the core measure to prevent postoperative recurrence. More importantly, arthroscopic surgery enables one-stage minimally invasive management of all concomitant lesions, including synovectomy, rotator cuff repair, and bone erosion debridement, which reduces surgical trauma, shortens the hospital stay, and accelerates postoperative rehabilitation.

For the management of concomitant lesions, individualized decision-making is essential. For the full-thickness rotator cuff tear in this case, we chose anatomical repair with a double-row suture bridge technique, which achieved robust tendon-bone fixation and good coverage of the footprint. This strategy not only restores the biomechanical function of the rotator cuff, but also closes the pathological channel between the glenohumeral joint and subacromial space, which may reduce the risk of PVNS recurrence in the subacromial space. For the small acromial bone erosion, we performed thorough debridement to remove all potentially invaded synovial tissue, rather than simple smoothing of the bone surface, to eliminate the residual recurrence nidus. For larger bone defects with structural instability, autologous bone grafting should be considered, as recommended by Ikuta et al. (11).

### Prognosis, recurrence risk, and long-term surveillance

Diffuse-type PVNS has a well-documented high local recurrence rate, ranging from 14% to 50% in previous literature (7, 12). The most critical risk factor for recurrence is incomplete resection of the pathological synovial tissue, and the presence of bone invasion is also associated with an increased recurrence risk. In this case, we performed thorough synovectomy of both the glenohumeral joint and subacromial space, as well as debridement of the bone erosion lesion, to minimize the risk of recurrence. The patient achieved significant pain relief and functional improvement at the 3-month and 5-month follow-ups, with the ASES score improving from 38.2 to 92.5, the VAS pain score decreasing from 6 to 1, and the Constant-Murley score improving from 30 to 88.

However, it must be emphasized that these results only reflect the early-stage functional recovery after surgery. The 5-month follow-up period without postoperative MRI is insufficient to confirm the structural healing of the rotator cuff tendon, nor can it rule out microscopic residual disease or late recurrence of PVNS. The majority of PVNS recurrences occur within 2–3 years after surgery, so long-term clinical and imaging surveillance is mandatory for this patient. We have scheduled annual clinical follow-up and shoulder MRI examinations for the patient to monitor rotator cuff healing and PVNS recurrence. Adjuvant radiotherapy will be recommended if there is any imaging sign of recurrence, as it has been shown to reduce the recurrence rate of diffuse PVNS after incomplete resection (3, 12). The patient declined adjuvant radiotherapy at the current stage due to satisfactory early outcomes, and this decision was fully respected with informed consent.

It must be explicitly stated that the current 5-month follow-up period is insufficient to draw any definitive conclusions regarding the long-term efficacy of this strategy, particularly with respect to PVNS recurrence (which typically occurs within 2–3 years postoperatively) and structural healing of the repaired rotator cuff tendon. Postoperative MRI has not yet been performed, which limits the assessment of residual microscopic disease. Therefore, the satisfactory early outcomes reported here should not be overinterpreted as evidence of cure or low recurrence risk.

### Study limitations

Several important limitations of this case report must be explicitly acknowledged. First, the core limitation of this study is the only 5-month short-term follow-up, with no postoperative shoulder MRI performed to date. This follow-up period is insufficient to assess the structural healing of the rotator cuff tendon, and cannot rule out microscopic residual PVNS or predict the long-term recurrence risk. The current results only reflect early postoperative functional recovery, and the long-term efficacy of the one-stage surgical strategy still needs to be verified by longer follow-up. Second, this study is a single case report, and the conclusions have limited extrapolation. The efficacy and safety of the one-stage combined arthroscopic strategy for this rare pathological triad still need to be validated by larger case series and multi-center prospective studies. Last, the patient declined adjuvant radiotherapy after surgery, so we cannot evaluate the impact of adjuvant radiotherapy on the recurrence risk of this case with PVNS-related bone erosion.

## Conclusion

This case reports a rare complete pathological triad of diffuse shoulder PVNS, full-thickness rotator cuff tear, and PVNS-induced acromial bone erosion, a combination that has not been previously documented in the literature, expanding the known clinical spectrum of locally aggressive shoulder PVNS. We have proposed a hypothesized pathological progression chain, based on intraoperative findings. This case reminds clinicians to maintain a high index of suspicion for PVNS in patients with chronic shoulder monoarthritis, even in the presence of typical rotator cuff tear signs. While the early functional outcomes are promising, the 5-month follow-up precludes any conclusions regarding PVNS recurrence or long-term tendon healing; therefore, cautious interpretation is warranted and rigorous long-term surveillance is mandatory.

## Data Availability

The raw data supporting the conclusions of this article will be made available by the authors, without undue reservation.
